# Particle Forming Amorphous Solid Dispersions: A Mechanistic Randomized Pharmacokinetic Study in Humans

**DOI:** 10.3390/pharmaceutics13030401

**Published:** 2021-03-17

**Authors:** Andreas Schittny, Samuel Waldner, Urs Duthaler, Alexander Vorobyev, Rimma Abramovich, Stephan Krähenbühl, Maxim Puchkov, Jörg Huwyler

**Affiliations:** 1 Division of Pharmaceutical Technology, Department of Pharmaceutical Sciences, University of Basel, 4056 Basel, Switzerland; andreas.schittny@unibas.ch (A.S.); s.waldner@unibas.ch (S.W.); maxim.puchkov@unibas.ch (M.P.); 2 Division of Clinical Pharmacology and Toxicology, Department of Biomedicine, University Hospital Basel and University of Basel, 4056 Basel, Switzerland; urs.duthaler@unibas.ch (U.D.); Stephan.Kraehenbuehl@usb.ch (S.K.); 3 Department of Pharmtechnology, Faculty of Advanced Training of Medical Workers, Peoples’ Friendship University of Russia (RUDN University), 117198 Moscow, Russia; alek_san2007@mail.ru (A.V.); abr-rimma@yandex.ru (R.A.); 4 Department of Clinical Research, University of Basel, 4056 Basel, Switzerland

**Keywords:** amorphous solid dispersions, bioavailability, poorly soluble drugs, clinical study, hot-melt extrusion

## Abstract

Amorphous solid dispersions (ASDs) are a promising drug-delivery strategy to overcome poor solubility through formulation. Currently, the understanding of drug absorption mechanisms from ASDs in humans is incomplete. Aiming to gain insights in this matter, we conducted a randomized cross-over design open-label clinical study (NCT03886766) with 16 healthy male volunteers in an ambulatory setting, using micro-dosed efavirenz as a model drug. In three phases, subjects were administered (1) solid ASD of efavirenz 50 mg or (2) dissolved ASD of efavirenz 50 mg or (3) a molecular solution of efavirenz 3 mg (non-ASD) as a control in block-randomized order. Endpoints were the pharmacokinetic profiles (efavirenz plasma concentration vs. time curves) and derived pharmacokinetic parameters thereof (*AUC_0–t_*, *C_max_*, *t_max_*, and *k_a_*). Results showed that the dissolved ASD (intervention 2) exhibited properties of a supersaturated solution (compared to aqueous solubility) with rapid and complete absorption of the drug from the drug-rich particles. All interventions showed similar *AUC_0–t_* and were well tolerated by subjects. The findings highlight the potential of particle forming ASDs as an advanced drug-delivery system for poorly soluble drugs and provide essential insights into underlying mechanisms of ASD functioning in humans, partially validating current conceptual models.

## 1. Introduction

Low oral bioavailability is a recurrent reason for drop-outs of poorly soluble drug candidates in preclinical and clinical stages of drug development [1,2]. Therefore, there is a need for reliable drug-delivery systems that can increase bioavailability. A promising candidate for such a drug-delivery system is a system based on amorphous solid dispersions (ASDs) [3]. In ASDs the active pharmaceutical ingredient (API) is delivered in its amorphous state, which is stabilized by a solid polymer matrix [4].

While it has been shown that the use of ASDs in oral drug delivery, in general, can significantly increase the bioavailability of different APIs in humans [5], there are also reports on in vivo examples, where bioavailability could not be increased [6]. Based on the mechanistic investigations, it was proposed that increased bioavailability results mainly from a temporary increase in the concentration of a molecularly dissolved drug, known as true supersaturation, by which the physicochemical limitations of poorly soluble APIs can be overcome [7]. The maximal supersaturation concentration thereby is the solubility in its amorphous state (amorphous solubility), above which amorphous liquid phase separation (ALPS) occurs, leading to the formation of drug-rich particles [8]. Polymers can stabilize the supersaturated state by preventing drug crystallization [9], but can also potentially reduce the concentration of molecularly dissolved API in favor of drug accumulation in the drug-rich phase of the particles [10]. Furthermore, surfactants can permanently solubilize and enclose drugs by forming micelles, limiting the API absorption due to a reduced concentration of molecularly dissolved drugs [11]. 

Most experimental data concerning the effects of ASDs on drug behavior originate from preclinical in vitro and in vivo studies. Clinical trials on ASDs were mainly performed in the context of commercial drug development, aiming to validate the formulation approach, without aiming to contribute to mechanistic understanding. Only a few clinical studies also investigated mechanistic aspects of increased bioavailability. One example is the study by Polster et al. [12]. In an artificial stomach-duodenum model, the authors characterized the mechanism of the increased bioavailability of LY2300599 as three steps: rapid supersaturation in the stomach, precipitation in the stomach into an amorphous solid, and redissolution of the amorphous solid in the duodenum with supersaturated drug concentration compared to the drug in crystalline form. A unique role was thereby attributed to the excipient meglumine. Othmann et al. [13] compared two ASD formulations (hot-melt extruded or spray-dried) of the compound ABT-102 with the solution of the API, each under fasting and non-fasting conditions. Both ASD formulations showed a significantly higher bioavailability than the solution formulation. The melt-extruded formulation did not show food effects, whereas the spray-dried formulation did. Also, Angi et al. [14] observed a significant food effect concerning the maximum plasma concentration in an ASD formulation of celecoxib. However, the impact of food on the time of maximum concentration was not discriminating due to a large plateau in the plasma concentration vs. time curves.

Despite the research effort on mechanisms of increased bioavailability through ASDs during the last decades, these mechanisms are far from being understood in every detail [5,7,15,16]. Furthermore, the translation from preclinical studies to clinical outcomes in humans has not been validated [17].

It was the aim of this clinical study to investigate the mechanisms and effects of a particle forming ASD on bioavailability in humans to gain an understanding of the complex absorption from ASDs in humans. To our best knowledge, there are currently neither documented data nor methods providing such insights. In this study, we used a model ASD formulation of efavirenz, composed of hydroxypropyl methylcellulose phthalate (HPMCP) as polymer and sucrose palmitate as well as polysorbate 80 as surfactants in a hot-melt extrusion process. Furthermore, we compared the study results with existing pharmacokinetic (PK) data on the marketed formulation (Stocrin^®^) [18].

## 2. Materials and Methods

### 2.1. Regulatory and Ethical Aspects

The clinical trial was conducted in adherence to Swiss law (Human Research Act, authorized by the ethics commission northeast and central Switzerland under the Swiss license number SNCTP000003251, registered also in the WHO recognized register clinicaltrials.gov under NCT03886766 and according to Good Clinical Practice (GCP) guidelines. All subjects documented their consent to participate in the study. Clinical samples were produced according to Good Manufacturing Practice (GMP) standards. The study was monitored by the Clinical Trial Unit of the Department of Clinical Research of the University of Basel.

### 2.2. Study Design and Population

The study population consisted of 16 healthy male volunteers between the age of 20 and 38 years. Exclusion criteria were the regular intake of medications less than two weeks before or during the study (exceptions without interaction potential with efavirenz could be granted by the investigator), smoking, and excessive alcohol consumption. The sample size of *n* = 16 was determined based on previous experience with cross-over design trials for 50 mg of efavirenz [18].

**Figure 1:**
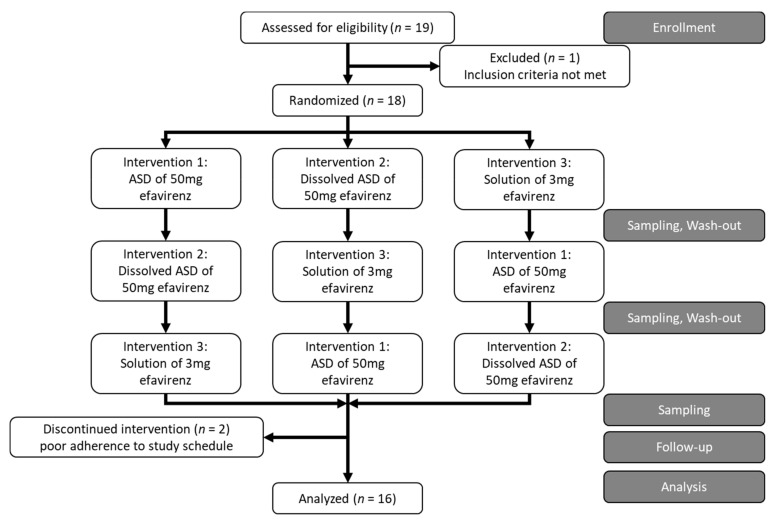
Study design according to the CONSORT Statement 2010 [20].

The study was designed as a single-center randomized 3-period cross-over study (Figure 1). Every subject ingested three different study products as a single dose (refer to Section 2.3). Study products were not blinded (open-label). To standardize for food effects, subjects have fasted overnight, standardized meals were then offered at specified time points, earliest at 4 h post-dose (voluntary consumption). Between the study interventions, a washout period of 14 to 21 days was maintained due to the long half-life of efavirenz of 52 to 76 h if administered as a single dose [19].Subjects were enrolled by the study investigators and were randomized according to the master randomization list (Appendix B) in the timely sequence of the subjects’ first intervention by the study investigators. Where dropouts occurred, the replacements were assigned to the same sequence as the dropout. The study was performed in the ambulatory study center of the Department of Clinical Research of the University of Basel. There were neither changes to the trial design or trial conduction nor changes in endpoints after the start of the trial.

### 2.3. Study Interventions

To assure study subject safety, a micro-dosing approach was chosen (3 or 50 mg of efavirenz). Subjects ingested three different study products in randomized order (1-2-3, 2-3-1, or 3-1-2):

- Intervention 1: ASD of efavirenz (50 mg) as a capsule with 500 mL buffer solution (see below),
- Intervention 2: Dissolved ASD of efavirenz (50 mg) in 500 mL buffer solution (see below), forming drug-rich particles (compare to Supplementary Material S2, Dynamic Light Scattering of intervention 2), and
- Intervention 3: Efavirenz (3 mg) solution in a 500 mL buffer solution (see below).

The buffer solution consisted of 10 mL of a medical sodium phosphate solution (Colophos^®^) added to 490 mL potable water (from the tap) resulting in a pH 6.3 solution with 6.62 mg/mL phosphates. The buffer solution was heated to 37 °C. ASDs were prepared through an optimized hot-melt extrusion process (for details refer to Section 2.4 as well as Appendix A, [21]). For intervention 2, the ASD of efavirenz (50 mg) was dissolved under standardized stirring conditions for 30 min. For intervention 3, 3 mL of a 1 mg/mL ethanol solution of efavirenz was diluted to 500 mL with buffer solution. For reasons of the chosen volume of 500 mL, please refer to Section 4.1.

### 2.4. Clinical ASD Study Samples

Capsules containing ASD of efavirenz for administration in the clinical study were produced as follows: The ASD was composed of efavirenz (22% *w*/*w*), hypromellose phthalate (HPMCP HP50, 62% *w*/*w*), sucrose palmitate (Surfhope^®^ SE D-1615, 13.5% *w*/*w*), and polysorbate (Tween^®^ 80, 2.5% *w*/*w*). Hot-melt extrusion was performed on a ZE9 9 mm mini extruder (Three-Tec, Seon, Switzerland). The resulting extrudate was milled and sieved (180 µm) before filling into gelatin capsules to the target dose of 50 mg of efavirenz, using mannitol and fumed silica as filler. The study medication was produced according to GMP guidelines. Please refer to Appendix A for more details on production and analytics.

### 2.5. Study Assessments and Outcomes

Blood samples were taken at −0.25 (predose sample), 0.25, 0.5, 0.75, 1, 2, 3, 4, 6, 8, 12, 24, 48, and 72 h post-dose. Plasma was produced by centrifugation and samples were stored at −20° C until sample analysis by LC-MS/MS (refer to Section 2.6). The primary study endpoints were the pharmacokinetic profiles of efavirenz plasma concentrations vs. time from all three study interventions. These profiles were used for further pharmacokinetic analysis and modeling (secondary endpoints, see Section 2.7). Safety outcomes were monitored according to the standard requirements for clinical trials.

### 2.6. Bioanalysis

The plasma concentration of efavirenz was quantified in March 2020 by LC-MS/MS consisting of a modular high-performance liquid chromatography system (Shimadzu, Kyoto, Japan) coupled to an API4000 Qtrap tandem mass spectrometer (AB Sciex, Framingham, MA, USA). Efavirenz was retained on a Kinetex C18 core-shell (2.6 µm, 50 × 2.1 mm, Phenomenex, Torrance, CA, USA) analytical column. Mobile phase A was water plus 0.1% acetic acid and mobile phase B was methanol. Aliquots of 50 µL plasma were extracted with 150 µL methanol containing 50 ng/mL efavirenz-d5, which was used as the internal standard. Extracts were centrifuged for 30 min at 3220× *g* and 10 °C. Afterward, 10 µL of supernatant was injected into the LC-MS/MS system at a flow rate of 0.6 mL/min and the column oven temperature of 45 °C. The following gradient program was used: 0–0.5 min, 5% B; 0.5–2 min, 5–95% B; 2–3 min, 95% B; 3–3.5 min, 5% B. In the first 0.5 min of each run, samples were online diluted with mobile Phase A, delivered by an additional pump, within a T-union installed in front of the analytical column.

Efavirenz and efavirenz-d5 reached the mass spectrometer after 2.1 min. Both analytes were charged in the negative mode by electrospray ionization and detected by multiple reaction monitoring. A mass transition of 314.0 → 243.8 and 319.0 → 247.8 was used for efavirenz and efavirenz-d5, respectively. Nitrogen was used as the curtain (10 L/min), collision (4 L/min), ion source 1 (60 L/min), and 2 (50 L/min) gas. The Ionspray voltage was set to −4200 V and the temperature of the interface was heated to 600 °C.

Efavirenz calibrations were prepared in plasma ranging from 0.5–1000 ng/mL. Moreover, quality control (QC) samples were prepared at the lower limit of quantification (LLOQ: 0.5 ng/mL), QC_Low_ (2.5 ng/mL), QC_Mid_ (25 ng/mL), QC_High_ (250 ng/mL), and the upper limit of quantification (ULOQ: 1000 ng/mL) level. An analytical run was accepted, if the mean accuracy was between 85 and 115% (LLOQ: 80–120%) and the precision ≤15% (LLOQ: ≤20%). Incurred sample reanalysis was performed for 149 out of 670 study samples (22.2%). The mean % difference was −4.2%, whereas only three samples showed a deviation of >20%, indicating that the measurements were reliable and reproducible.

### 2.7. Pharmacokinetics and Modeling

Baseline correction was applied to plasma samples where efavirenz plasma concentrations above the lower limit of detection were measured in the predose (−0.25 h) samples, indicating incomplete washout of the previously administered dose. The efavirenz plasma concentration of the predose sample was subtracted from every further acquired sample according to the first-order elimination kinetics extrapolation (elimination rate constant obtained by non-compartmental analysis, an average of all three study arms of the individual subjects).

The area under the curve *AUC_0–t_*, maximum concentration *C_max_*, and the time to maximum concentration *t_max_* were retrieved from the standard non-compartmental analysis. The absorption constant *k_a_* was obtained from a two-compartmental fitting performed in Mathematica version 12.1.0.0 (2020, Wolfram Research Inc., Champaign, IL, USA). Fitting details are given in Appendix C.

In an attempt to interpret obtained plasma concentration—time curves in terms of in vivo intestinal dissolution profiles, a physiologically based pharmacokinetic (PBPK) model was established and fitted to the obtained PK-profiles (Supplementary Material S1, PBPK Modeling: 1 Background, 2 Methods with Table S1**.** Constant PBPK parameters, Table S2. PBPK fitting parameters, Table S3. Weibull model fitting parameters).

### 2.8. Biostatistics

Statistical analysis was performed for the complete sample of 16 subjects. All data were tested for a normal distribution using the Shapiro–Wilk test [22]. Pharmacokinetic parameters retrieved from the different study interventions were compared in a one-way repeated measures ANOVA analysis at a 0.05 level of significance. The sphericity assumption was tested using Mauchly’s test [23]. Sphericity assumption violations were corrected for using the Greenhouse-Geisser correction (for Greenhouse-Geisser Epsilon < 0.75) or Huynh-Feldt (for Greenhouse-Geisser Epsilon ≥ 0.75) [24]. Effect size (ω^2^) was calculated separately in Microsoft Excel version 2016 (Microsoft, Redmond, WA, USA). Pairwise post hoc analysis was performed using the Bonferroni method. Study results were compared with existing data using one-way ANOVA at a 0.05 level of significance. The equal variance assumption was tested according to Levene [25]. Equal variance assumption violations were accounted for by calculating the corrected F-ratio according to Welch [26] separately in Excel. Pairwise post hoc analysis was performed using a post hoc Bonferroni test considering *p* < 0.05 as statistically significant. The main analysis was performed in Origin Pro 2018 version b9.5.195 (2018, Origin Lab Corporation, Northampton, MA, USA).

## 3. Results

### 3.1. Trial Conduction and Subjects

In total, 18 subjects were enrolled and 16 subjects completed the study. The two dropouts were caused by non-compliance with the study schedule. All 16 subjects that completed the study were included in the analysis. The recruitment was started in August 2019, the first intervention was performed on 16 September 2019, and the last visit by a subject was on 19 March 2020. The trial was stopped upon completion, i.e., when all study phases were completed by 16 subjects. Table 1 shows the baseline characteristics of the subjects included.

### 3.2. Pharmacokinetic Analysis

**Figure 2:**
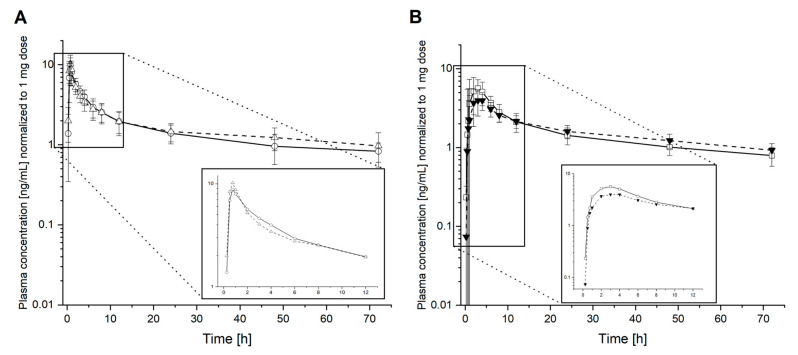
Normalized pharmacokinetic profiles. Efavirenz plasma concentrations vs. time curves of efavirenz administered in a dissolved state (**A**) in intervention 2 (dissolved amorphous solid dispersions (ASD) of efavirenz 50 mg, ○) and intervention 3 (solution of efavirenz 3 mg, △); as well as efavirenz administered in a solid-state (**B**) in intervention 1 (ASD of efavirenz 50 mg, □) and the marketed formulation (50 mg, ▼) [18] in B are shown. Error bars: standard deviation.

Figure 2 shows the obtained pharmacokinetic profiles of efavirenz for the different interventions as well as the marketed formulation [18] normalized to 1 mg of dose. While intervention 2 (dissolved ASD of efavirenz 50 mg) and intervention 3 (solution of efavirenz 3 mg) showed sharp and early concentration peaks, intervention 1 (ASD of efavirenz 50 mg) and the existing data on the marketed formulation do not reveal a pronounced extremum in plasma concentration. Based on the obtained profiles from all subjects, an effect of the enterohepatic recirculation, which can result in additional peaks, could not be observed as described in some subjects [27].

**Figure 3:**
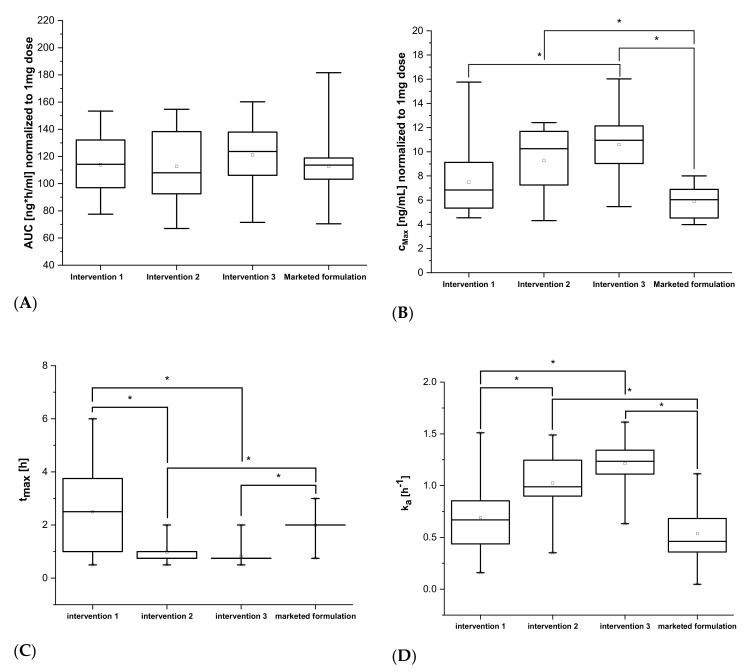
Box plots of normalized pharmacokinetic parameters retrieved from the non-compartmental analysis. The area under the curve *AUC_0–t_* (**A**), maximum concentration *C_max_* (**B**) and the time of maximum concentration *t_max_* (**C**) as well as from two-compartment analysis, i.e., absorption constant *k_a_* (**D**) for intervention 1 (ASD of efavirenz 50 mg), intervention 2 (dissolved ASD of efavirenz 50 mg), intervention 3 (solution of efavirenz 3 mg), and the marketed formulation 50 mg [18] are shown. *AUC_0–t_* and *C_max_* are normalized to a dose of 1 mg efavirenz. Boxes show the interquartile range with the median, whiskers show the 5th (low) and 95th (high) percentile, and the hollow squares the mean values. A statistically significant difference (*p* < 0.05, Bonferroni test) is indicated by *.

Figure 3 shows the derived pharmacokinetic parameters, the data for *AUC_0–t_* and *C_max_* are normalized to 1 mg of dose. Corresponding means with 95%—CIs are shown in Table 2. All interventions as well as the non-cross-over comparison to the marketed formulation showed comparable areas under the curve *AUC_0–t_*. Intervention 3 (solution of efavirenz 3 mg) showed the highest maximum concentration *C_max_* and the shortest time to reach maximum concentration *t_max_*, with a statistically significant difference to both intervention 1 (ASD of efavirenz 50 mg) and the marketed formulation (*p* < 0.05). Furthermore, the comparison between intervention 3 and intervention 2 (dissolved ASD of efavirenz 50 mg) regarding *C_max_* and *t_max_* showed only small, statistically insignificant differences (*p* > 0.05). The same applies to the comparison of intervention 1 (ASD of efavirenz 50 mg) and the marketed formulation (no statically significant differences). The absorption constant *k_a_* results showed statistically significant differences between the liquid (intervention 2 and 3) and the solid formulations (intervention 1 and marketed formulation). For detailed statistical results refer to Appendix D.

Results on modeled in vivo dissolution profiles by PBPK model fitting are shown in the Supplementary Materials.

### 3.3. Safety Outcomes

The interventions were well tolerated by the subjects; no serious adverse events were reported. One adverse event with a likely correlation to the study interventions was mild irritation of the oral and laryngopharyngeal mucosa after intervention 2 (dissolved ASD of efavirenz 50 mg), which could be successfully treated and prevented by rinsing of the mouth with water after complete ingestion of the solution. A single adverse event with a possible correlation to the study drug efavirenz was a mild and localized skin rash observed starting approximately 3 weeks after completion of the study, which resolved without sequel upon topical therapy with prednicarbate.

## 4. Discussion

### 4.1. Study Design and Rationale for Interventions

**Figure 4:**
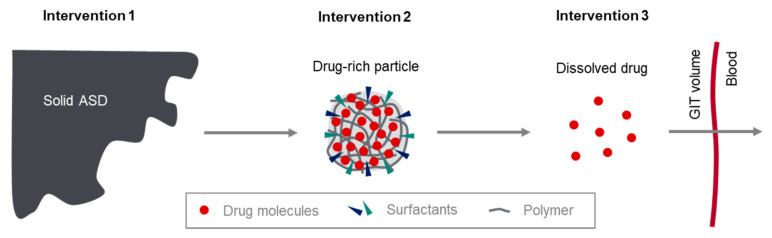
Conceptual model describing the drug release from ASDs, the formation of drug-rich particles, and intestinal absorption of the molecularly dissolved drug. The solid ASD dissolves into drug-rich particles (presumably composed of drug, polymer, and surfactants), from which molecularly dissolved drug is liberated and absorbed (adapted from [28], Taylor & Francis Group, 2019).

The design of the present study and its interventions (study arms) was based on a mechanistic model describing drug release from ASDs and subsequent absorption in the gastrointestinal tract. This conceptual model consists of three different phases (Figure 4), which can be summarized as follows:

1. The dissolution from solid ASD into the dissolved state of the ASD, showing drug-rich particles;
2. Drug liberation from the dissolved state (phase 1) of the ASD to molecularly dissolved drug;
3. Absorption of the molecularly dissolved drug in the gastrointestinal tract.

In the present study, three different drug formulations (study intervention 1–3) were tested, allowing for the investigation of the transition between the three conceptual stages outlined in Figure 4 in humans. Intervention 3 was used to study intestinal absorption of the molecularly dissolved drug, intervention 2 represents the drug in form of drug-rich particles, and intervention 1 is the solid ASD.

It should be noted that the formation of the drug-rich particles is concentration-dependent and that the solubility of efavirenz in water is limited. Therefore, it was not possible to administer the same doses in all interventions. The volume of the drinking solution was maximized to 500 mL to maximize the dose of efavirenz that could be delivered in a solution (3 mg). This was necessary to guarantee sufficient plasma levels for concentration measurement. The dose of efavirenz delivered as ASD (50 mg) was increased high enough to guarantee the formation of a drug-rich particle but as close as possible to the comparative dose of 3 mg, even though literature data suggest that efavirenz has linear pharmacokinetics over an extended dose range [19,29]. Furthermore, saturation effects leading to nonlinear pharmacokinetics are unlikely at low doses. Linearity in the dose range of this study is supported by the almost identical pharmacokinetic profile of intervention 2 (dissolved ASD of efavirenz 50 mg) and intervention 3 (efavirenz solution 3 mg). Since no intravenous formulation of efavirenz is available [27], as is the case for many poorly soluble drugs, the low dose oral solution seems to be the best comparator for study formulation effects. As the therapeutic dose of efavirenz is 600 mg daily, the doses used in the present study (3 and 50 mg) can be considered as a micro-dosing approach. Since a possible increase in efavirenz plasma concentrations compared to the marketed formulations could not be excluded in advance, the micro-dosing approach mitigates the risk of non-tolerable plasma levels in subjects.

It is exceedingly difficult to identify the reasons for reported inefficiencies of ASDs in animal or clinical studies. Our approach made it possible to investigate the mechanisms of increased bioavailability from ASDs in humans with a comparably simple and safe clinical study. The conceptual phases of our mechanistic model can be understood as a series of critical steps covering drug release from the formulation and drug absorption. To the best of our knowledge, this is the first study using such an approach to identify mechanisms limiting or enhancing the performance of ASDs.

The comparison of the novel efavirenz ASD formulation to existing formulations was not the primary aim of this study and was therefore not implemented as an additional study arm within the cross-over design. However, a comparison of our study results with existing data of a marketed formulation of efavirenz 50 mg gives insights into the performance of the novel formulation compared to a benchmark product.

Efavirenz has been used frequently for the research on ASDs [30,31,32] since it is classified by some authors as a BCS class II drug [33], i.e., with poor solubility but high permeability. Due to the lack of an intravenous formulation, no human data on the absolute bioavailability of efavirenz [27] are available. Data in animals might hint toward a poor absolute bioavailability (16% in rats and 42% in monkeys [19]); however, even for the more reliable data for bioavailability from monkeys [34], a direct translation of animal data to humans is questionable. Various authors state the absolute bioavailability in humans to be 40–45%, but without referencing original data in humans. However, looking at the obtained results, it seems that efavirenz might be subject to a ceiling effect. Indeed, all interventions as well as the marked formulation show comparable *AUC_0–t_*. It is therefore tempting to speculate that the absolute bioavailability of efavirenz might be higher than observed in preclinical studies, making it challenging to increase the bioavailability further. This is also suggested by the rapid absorption (high *k_a_*), indicating that the absorption of efavirenz might be complete. On the other hand, some authors proposed that efavirenz is less permeable than commonly described in the literature [35], which could reduce the sensitivity to detect formulation effects on the pharmacokinetics of efavirenz (*t_max_* and *k_a_* values). However, taking into account the linear pharmacokinetics and the distinctly different values for *k_a_* observed in the different interventions in this study, it is suggested that permeability does not limit the bioavailability (*AUC_0–t_*) of efavirenz. Regarding the metabolism of efavirenz, it should be noted that in the present study only plasma concentrations of the parent drug were determined, as drug elimination was not a focus of this study. Furthermore, the metabolites (mainly 8-hydroxy-efavirenz through CYP2B6 but also 7-hydroxy-efavirenz through CYP2A6 [36]) are not relevant pharmacologically [37].

The sample size in this study was determined based on experience with previous pharmacokinetic studies. Here, the low within-subject CV of 6.9% (*AUC_0–24 h_* ratio between cross-over interventions) indicates that the sample size of 16 subjects is large enough to differentiate between interventions [18]. Furthermore, to compare the existing data of a marketed formulation to our study data, an equal sample size maximizes the ANOVA robustness. For the chosen sample size, normality violations were observed in some cases, which, despite the robustness of variance analysis regarding these violations, represent a limitation in the statistical analysis. For further details, refer to Appendix D. The wash-out period of at least 14 days was chosen to keep the inclusion time of study subjects short. In some subjects, baseline corrections were necessary due to the long half-life of efavirenz. Quantitatively, the corrections were negligible and should therefore not have influenced our interpretations of the results.

### 4.2. Effects of ASD on the Bioavailability of Efavirenz in Humans

Looking at the pharmacokinetic analysis, it is striking that intervention 2 (dissolved ASD of efavirenz 50 mg) behaved almost identically to intervention 3 (solution of efavirenz 3 mg). This indicates that even though intervention 2 consisted of a supersaturated aqueous solution containing drug-rich particles as shown earlier by our group [21], it pharmacokinetically behaved like a solution. Under the assumption of passive absorption of molecularly dissolved efavirenz, it therefore can be deduced that the dissolved drug concentration in the intestine (the site of absorption) of intervention 2 was proportionally higher than in intervention 3 (by a factor of 50/3 = 16.7). The drug concentration in intervention 2 (50 mg/500 mL) exceeded the aqueous solubility of efavirenz (approximately 10 mg/L [35]) by far, even when taking into account the possibility of larger volumes of gastrointestinal fluids being present before administration. We could therefore show for the first time in humans, that drug-rich particles resulting from ASDs are an efficient oral drug-delivery system with a rapid and complete transformation of the drug into the systemic circulation.

Regarding the equilibria of the different states of the drug (molecularly dissolved, amorphous liquid phase separation, and crystalline), the solution-like behavior indicates the absence of any hindrance to drug absorption caused by the delivery system, even if part of the drug is expected to be in the drug-rich particles initially. Based on our review [28], these results support the hypothesis that (1) crystallization of drug could be prevented in the investigated formulation, (2) there was no permanent (irreversible) solubilization of drug into micelles which would prevent drug absorption, and (3) the polymeric particles did not reduce the intestinal concentration of molecularly dissolved drug in the chosen study setting. These are important prerequisites for an ASD to function as a bioavailability-increasing drug-delivery system. Drug delivery in the form of drug-rich particles seems to facilitate a fast and efficient drug absorption and to reduce the previously modeled late efavirenz absorption from standard formulations in distant intestinal parts [38].

The question arises to which degree bile salts might influence the ASDs performance in the present study. Subjects have fasted, therefore, the aqueous drinking solution is expected to trigger only a partial gall bladder emptying [39]. However, it was reported that bile salts significantly increase the solubility of efavirenz from 10 to 194 mg/L in fasted simulated intestinal fluid containing bile salts (FaSSIF) [40]. Further, no food effect on bioavailability at therapeutic doses was observed at a dose for 100 mg [40], indicating that bioavailability could be high at low doses, which would explain the comparable *AUC_0–t_* for the different interventions as well as for the marketed formulation. For the rationale of the choice of the dose regimen used in this study, refer to Section 4.1. Based on these results, dose-escalation studies would be necessary to shed more light on the factors influencing the bioavailability of efavirenz in humans.

As intervention 2 (dissolved ASD of efavirenz 50 mg) showed a complete and fast absorption, the delayed absorption in intervention 1 (ASD of efavirenz 50 mg) is most likely caused by the dissolution of the solid ASD to drug-rich particles. This process was slightly faster and more efficient than dissolution from the marketed tablet (refer to Section 4.4). This is a positive finding in the context of improving human bioavailability, showing that the ASD formulation approach can result in an efficient dissolution process. Regarding dissolution mechanisms from ASDs into drug-rich particles in humans, detailed conclusions are not possible based on this study.

### 4.3. Translational Aspects

Based on our previously published preclinical results of the ASD formulation used in this study [21], results of in vitro dissolution tests and the animal PK study do not translate well into humans. This underlines the difficulty of extrapolation from in vitro results and between species regarding bioavailability. Even for conventional formulations, only a poor link between animal and human oral bioavailability could be established [34]. It can be assumed that for more complex formulations such as ASDs, translation is even more complex. Therefore, more clinical trials might be advisable to advance novel or more complex formulation strategies.

### 4.4. ASD Formulation

The presented data from the novel ASD formulation utilized in the present study were compared to an existing marketed formulation [18]. Based on the obtained *t_max_* and *C_max_* values, the dissolution process of the ASD formulation was not different from the marketed formulation based on the statistics performed.

Based on *AUC_0–t_* values (as an indicator for bioavailability), the ASD formulation of efavirenz performed as well as the marketed formulation. It is worthwhile to note, that the marketed tablet formulation we used for our comparisons contains solubility enhancers (sodium lauryl sulfate) [41] and therefore has a higher bioavailability than the marked solution in triglycerides [19]. Overall, the novel ASD formulation used in this study compared favorably to the marketed product regarding pharmacokinetic performance. Furthermore, this can be regarded as a positive example for the use of the surfactants sucrose palmitate and polysorbate 80 in an ASD formulation in humans. Further comparative investigations would be necessary to elucidate the isolated effects of the surfactants and if their effect would align with observations made in preclinical development [21]. A limitation of this comparison is that a high to complete bioavailability of efavirenz at the doses used in this study cannot be excluded.

A limitation of this comparison is the non-crossover design (different subject groups) and a relatively small sample size. Furthermore, the clinical procedure for the administration of the marked formulation was slightly different from the one in this study: For the marketed formulation, subjects ingested the tablet with only 240 mL of water compared to 500 mL of buffer in our study.

In an attempt to shed light on the dissolution behavior of the formulation in vivo, a PBPK model was fitted to the experimentally measured PK profiles in order to extract simulated in vivo dissolution curves. Their discussion including limitations are shown in Supplementary Material S1, PBPK Modeling: 4 Discussion.

From the pharmaceutics point of view, an unaltered implementation of this novel formulation as a therapeutic product would be challenging due to the low drug load of 22% (*w*/*w*), resulting in a high pill burden. A dose-escalation study would be necessary to investigate the possible increase in bioavailability, and therefore reduction of dose, at therapeutic doses.

## 5. Conclusions

In this pharmacokinetic study, we mechanistically investigated the effects of a novel, particle-forming amorphous solid dispersion (ASD) on the relative bioavailability using efavirenz as a micro-dosed model drug. We could show that drug absorption from drug-rich particles, formed upon the dissolution of the ASD, was fast and complete in humans. These findings confirm conceptual models of drug release from ASDs and subsequent intestinal drug absorption; drug-rich particles from the ASD seemed to prevent drug crystallization as well as permanent solubilization of drug into micelles. Overall, a molecularly dissolved drug concentration increase beyond aqueous saturation concentration could be deduced for the intestinal tract. These are important prerequisites for ASDs to function as bioavailability-increasing drug-delivery systems. Besides, our study yields essential insights into the so far poorly understood behavior of ASDs in humans. The specific efavirenz formulation developed for our study compared well with existing data on a marketed efavirenz formulation, underlining the potential of ASDs as an advanced drug-delivery system for increasing bioavailability of poorly soluble drugs. We propose that the micro-dosing approach used in this study is a safe and cost-efficient method for early investigations of ASD formulations and possibly of other advanced drug-delivery systems.

## Figures and Tables

**Table 1:** Baseline characteristics of the study population (healthy male volunteers).

Characteristics	Mean (SD)
Age [years]	28.9 (5.4)
BMI [kg·m^−2^]	25.1 (2.6)
Resting heart rate [min^−1^]	66.7 (10.1)
Systolic blood pressure [mmHg]	123.4 (11.9)
Diastolic blood pressure [mmHg]	69.1 (7.7)

**Table 2:** Normalized Pharmacokinetic parameters mean (95%—CI) for intervention 1 (ASD of efavirenz 50 mg), intervention 2 (dissolved ASD of efavirenz 50 mg), intervention 3 (solution of efavirenz 3 mg), and the marketed formulation 50 mg [18]. This summary is provided under CONSORT guidelines [20].

PK-Parameter	Intervention 1	Intervention 2	Intervention 3	Marketed Formulation
*AUC_0–t_* [ng·h/mL]	113.9	112.7	121.2	112.8
(norm. to 1 mg)	(101.9–125.9)	(98.6–126.8)	(107.5–134.8)	(99.7–125.9)
*C_max_* [ng/mL]	7.5	9.3	10.6	5.9
(norm. to 1 mg)	(5.9–9.1)	(7.8–10.8)	(9.2–12.0)	(5.2–6.6)
*t_max_* [h]	2.4	1.0	0.8	2.0
	(1.5–3.3)	(0.7–1.3)	(0.6–1.0)	(1.6–2.4)
*k_a_* [h^−1^]	0.7	1.0	1.2	0.5
	(0.5–0.9)	(0.9–1.2)	(1.1–1.3)	(0.4–0.7)

## Data Availability

Study data is available on request from the corresponding author. The data are not publicly available due to the documentation on paper in accordance with the study protocol section 12.2.2.

## Supplementary Materials

The following are available online at https://www.mdpi.com/1999-4923/13/3/401/s1: Supplementary Material S1, PBPK Modeling: 1 Background, 2 Methods (Table S1. Constant PBPK parameters, Table S2. PBPK fitting parameters, Table S3. Weibull model fitting parameters), 3 Results (Figure S1. Concentration vs. time curve of dissolved efavirenz in the gastrointestinal tract), 4 Discussion. Supplementary Material S2, Dynamic Light Scattering of intervention 2 (dissolved ASD of evafirenz 50 mg).

## Appendix A. Formulation Production

The ASD composed according to Table A1 was produced according to the settings in Table A2. The resulting extrudate was milled, sieved through a 180 µm sieve, and filled into gelatin capsules using mannitol and fumed silica as filler to 50 mg efavirenz per capsule. The efavirenz solution was produced by dissolving pure efavirenz in ethanol to 1 mg/mL. Formulations were analyzed according to Table A3. All clinical samples were produced according to GMP rules.

**Table A1:** ASD composition.

Solid Compound	Weight Percent [%]
Efavirenz	22
HPMCP HP50	62
Sucrose palmitate (Surfhope^®^ SE D-1615)	13.5
Polysorbate (Tween^®^ 80)	2.5

**Table A2:** ASD production settings on a Three-Tec (Seon, Switzerland) ZE9 9 mm mini extruder.

Parameter	Setting
Entry Zone	Water cooling
Zone 1 (closest to the entry)	0 °C (no heating)
Zone 2	130 °C
Zone 3	140 °C
Zone 4	145 °C
Zone 5 (closest to exit)	150 °C
Screw speed	150 rpm
Solid Feed rate	Approx. 0.5 g/min ^a^
Liquid feed rate	Approx. 12.5 mg/min = 11.63 µL/min ^b^
Screw	Standard, no kneading disks
Die plate	1 mm

^a^ Calibrated for every process. ^b^ Calculated according to the solid feed according to weight percentages in the composition.

**Table A3:** ASD formulation analytics.

Test	Formulation	Method	Specification
Content	ASD, solution	Quantification by HPLC	According to Ph. Eur. 2.9.6.
Content uniformity	ASD	Quantification by HPLC	According to Ph. Eur. 2.9.40.
Dissolution	ASD	According to Ph. Eur. 2.9.3, quantification by HPLC	At least 90% release after 20 min, an average of 6 samples
Amorphous state	ASD	XRPD	No crystallinity peaks

## Appendix B. Randomization List

The randomization master list (Table A4) was generated by the sponsor-investigator in two steps: (1) random determination, which treatment sequence was used six times (other sequences five times) and (2) random scrambling of the treatment sequences for the participant numbers according to the frequencies determined in (1). The generation of the master randomization list, the enrollment, and assignments of the subjects was performed by the sponsor-investigator.

**Table A4:** Randomization list.

Subject Number	Study Formulation Administration		
	Phase A	Phase B	Phase C
1	2	3	1
2	3	1	2
3	2	3	1
4	2	3	1
5	1	2	3
6	2	3	1
7	1	2	3
8	3	1	2
9	1	2	3
10	3	1	2
11	3	1	2
12	3	1	2
13	2	3	1
14	2	3	1
15	1	2	3
16	1	2	3

## Appendix C. Compartment Model Fitting

Fitting of the pharmacokinetic data to the extravascular two-compartment model was done using Mathematica’s (version 12.1.0.0, Wolfram Research Inc., Champaign, USA) NonlinearModelFit function. All four rate constants (*K_a_*, *K_e_*, *K_central>peripheral_*, and *K_peripheral>central_*), as well as the distribution volume VD, were constrained to be greater than zero. Manual adaption of fitting parameters was necessary in rare cases to get reasonable results: if the fitted parameters were at the constraint boundary i.e., zero, the constraint for that parameter was increased from 0 h^−1^ to 0.00001 h^−1^. Where the rate constant *K_peripheral>central_* was unrealistically high (2 to 50 h^−1^), it was additionally constrained to be lower than 2 h^−1^. For datasets with apparent lag time, the data were truncated manually to remove the lag time.

## Appendix D. Statistical Details

Detailed results of repeated measures ANOVA and standard ANOVA are shown in Table A5 and Table A6. The assumption of normality was violated according to the Shapiro–Wilk test for the *AUC_0–t_* data of the marketed formulation, for the *C_max_* data of intervention 1 (ASD of efavirenz 50 mg), for the *t_max_* data of intervention 2 (dissolved ASD of efavirenz 50 mg), intervention 3 (solution of efavirenz 3 mg), and the marketed formulation. This is reasonable as the sample size was small and some of the data, especially the *t_max_* data, contained a lot of repeated values. As analyses of variance are relatively robust against violations of the normality assumption as long as the sample sizes are equal and two-tailed tests are used [42,43], their output can still be expected to be relevant.

**Table A5:** Detailed repeated measures ANOVA results (comparisons within cross-over design study).

Parameter	Mauchly’s Test of Sphericity		Greenhouse-Geisser Correction	Repeated Measures ANOVA Results		
*AUC_0–t_*	χ^2^(2) = 7.59	*p* < 0.05	applied (ε = 0.69)	F(1.39, 19.41) = 2.42	*p* > 0.05	ω^2^ = 0.011
*C_max_*	χ^2^(2) = 0.85	*p* > 0.05	n/a	F(2, 30) = 6.01	*p* < 0.05	ω^2^ = 0.146
*t_max_*	χ^2^(2) = 23.26	*p* < 0.05	applied (ε = 0.55)	F(1.10, 24.98) = 14.30	*p* < 0.05	ω^2^ = 0.368
*k_a_*	χ^2^(2) = 4.61	*p* > 0.05	n/a	F(2, 30) = 12.15	*p* < 0.05	ω^2^ = 0.239

**Table A6:** Detailed ANOVA results (comparison to existing data on the marketed formulation with a non-cross-over design).

Parameter	Levene’s Test of Equal Variance		ANOVA Results		
*AUC_0–t_*	F(3, 60) = 0.73	*p* > 0.05	F(3, 60) = 0.73	*p* > 0.05	ω^2^ = −0.027
*C_max_*	F(3, 60) = 2.92	*p* < 0.05	F_Welch_(3, 31.0) = 16.23	*p* < 0.05	ω^2^ = 0.312
*t_max_*	F(3, 60) = 12.56	*p* < 0.05	F_Welch_(3, 31.0) = 17.35	*p* < 0.05	ω^2^ = 0.361
*k_a_*	F(3, 60) = 0.70	*p* > 0.05	F(3, 60) = 17.23	*p* < 0.05	ω^2^ = 0.210
