# Supplementary material for: Particle Forming Amorphous Solid Dispersions: A Mechanistic Randomized Pharmacokinetic Study in Humans
Source: Pharmaceutics. 2021 Mar 17;13(3):401. doi: 10.3390/pharmaceutics13030401 (PMC8003007; doi:10.3390/pharmaceutics13030401)
Supplement: Supplementary file 1 [file pharmaceutics-13-00401-s001.pdf]

pharmaceutics

Supplementary Material: Particle Forming Amorphous Solid Dispersions: A Mechanistic Randomized Pharmacokinetic Study in Humans

Andreas Schittny, Samuel Waldner, Urs Duthaler, Alexander Vorobyev, Rimma Abramovich, Stephan Krähenbühl, Maxim Puchkov and Jörg Huwyler

Citation: Schittny, A.; Waldner, S.; Duthaler, U.; Vorobyev, A.; Abramovich, R.; Krähenbühl, S.; Puchkov, M.; Huwyler, J. Particle Forming Amorphous Solid Dispersions: A Mechanistic Randomized Pharmacokinetic Study in Humans. *Pharmaceutics* **2021**, *13*, 401. https://doi.org/10.3390/pharmaceutics13030401

Publisher's Note: MDPI stays neutral with regard to jurisdictional claims in published maps and institutional affiliations.

Copyright: © 2021 by the authors. Submitted for possible open access publication under the terms and conditions of the Creative Commons Attribution (CC BY) license (http://creativecommons.org/licenses/by/4.0/).

Supplementary Material S1. PBPK Modeling

1. Background

Aiming to understand intestinal dissolution of the investigated formulations, a physiologically based pharmacokinetic (PBPK) model was established and fitted to the obtained PK-profiles to extract simulated in vivo dissolution curves. With this, an attempt was made to bridge the limited feasibility of interventions in the clinical study to the needs of in vivo dissolution results for rational formulation development.

2. Methods

The PBPK-modeling was performed using the open-source PBPK software PK-Sim Version 8 (2020, Open Systems Pharmacology). In the first step, the PBPK-model parameters were fitted to the experimentally measured PK profile of intervention 3 (solution of efavirenz 3 mg) to calibrate the model. Where available, published or calculated data were used as starting values for the fitting. In a second step, the obtained parameters of the solution (intervention 3) were kept constant and Weibull dissolution [1] parameters were fitted to the experimentally measured PK profiles of intervention 1 (ASD of efavirenz 50 mg), intervention 2 (dissolved ASD of efavirenz 50 mg), and the marketed formulation. Details on PBPK model fitting are given in Table S1 (constant PBPK model parameters for all interventions and the marketed formulation), Table S2 (fitting parameters for intervention 3 and subsequently intervention 1, 2, and the marketed formulation), and Table S3 (fitting parameters of Weibull functions for intervention 1, 2, and the marketed formulation).

Table S1. Constant PBPK parameters.

Parameter	Setting	Source
Individual	European (prebuilt in PK-Sim)	[2]
Fraction unbound	3%	[3]
Molecular weight	325.68 g/mol	[3]
Solubility (FaSSIF)	0.19 mg/mL	[4]
Partition coefficients calculating method	PK-Sim Standard	-
Cellular permeabilities calculation method	PK-Sim Standard	-

Table S2. PBPK fitting parameters.

Parameter	Fitted for Intervention	Identified Value	Starting Value	Source	Minimum Value	Maximum Value
Lipophilicity	1	3.24 log units	4.02 log units	[3]	2.00	6.00
Permeability	1	0.13 cm/min	1.48 cm/min	[a]	0.0148 cm/min	148 cm/min
Renal plasma clearance	1	0.58 mL/min/kg	0 mL/min/kg	-	0 mL/min/kg	20 mL/min/kg
Intestinal Permeability	1	$1.20 \cdot 10^{-4}$ cm/min	$3.09 \cdot 10^{-3}$ cm/min	[a]	$3.09 \cdot 10^{-3}$ cm/min	0.309 cm/min
Weibull dissolution time	2, 3, marketed	[b]	1	-	0.001	10
Weibull dissolution shape	2, 3, marketed	[b]	0.001 min	-	0.001 min	360 min
Weibull lag time	2, 3, marketed	[b]	0.001 min	-	0.001 min	360 min

[a] Predicted by PK-Sim®, [b] Listed in **Table S3.**

Table S3. Weibull model fitting parameters.

Parameter	Dissolution Shape	Dissolution Time [min]	Lag Time [min]
Intervention 1 (ASD)	0.71	65.05	0.001
Intervention 2 (dissolved ASD)	0.84	1.51	0.00
Marketed formulation	0.61	87.56	3.03

A Monte Carlo algorithm implemented in PK-Sim was used for model fitting. Due to the limited number of sampling points, the Runge's phenomenon (oscillation of the fitted function around the experimentally measured data points) was observed when modeling enterohepatic recirculation. Therefore, enterohepatic recirculation was neglected in the model, as also described in the literature [5].

3. Results

Figure S1 shows the modeled dissolved concentration vs. time curves of efavirenz in the gastrointestinal tract based on PBPK model fitting of experimentally measured plasma concentration vs. time curves. For intervention 2 (dissolved ASD of efavirenz 50 mg), an immediate dissolution was modeled. In the case of intervention 1 (ASD of efavirenz 50 mg), the model showed a complete dissolution within 40 h, while for the marketed formulation (efavirenz 50 mg) an incomplete dissolution up to 80% was modeled based on results of experimentally measured plasma concentrations over 72 h.

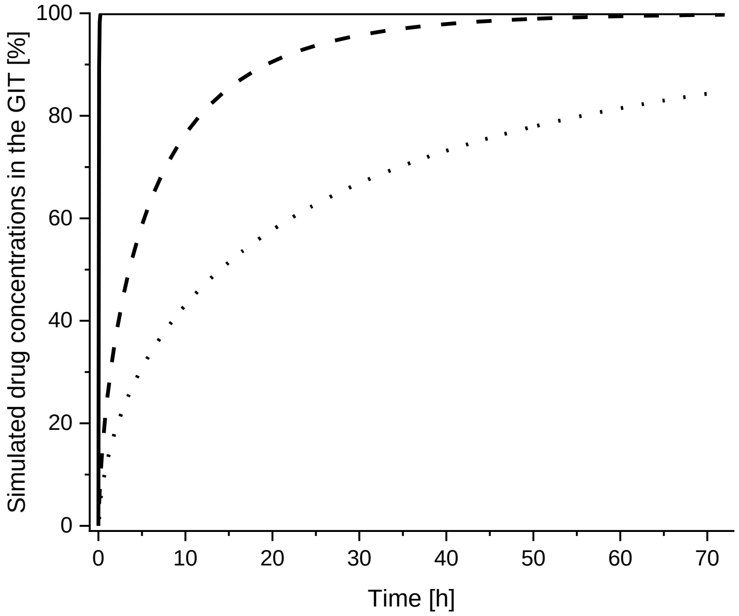

Figure S1. Concentration vs. time curves of dissolved efavirenz in the gastrointestinal tract. Modeled curves based on fitting a PBPK model to the experimentally measured PK profiles of intervention 1 (ASD of efavirenz 50 mg, dashed line), intervention 2 (dissolved ASD of efavirenz 50 mg, solid line), and the marketed formulation (efavirenz 50 mg, dotted line). The model was previously calibrated based on experimentally measured PK profiles of intervention 3 (solution of efavirenz 3 mg).

4. Discussion

The model suggests that intervention 2 (dissolved ASD of efavirenz 50 mg) was instantly and completely delivered through the intestinal wall (as compared to intervention 3, the solution of efavirenz, to which the model was calibrated prior to fitting the dissolution curves in Figure S1). This highlights that through the dissolved ASD formulation, a higher dose (50 mg) compared to the pure efavirenz solution (3 mg, dose limited by solubility) was delivered with the same pharmacokinetic properties. With respect to the function of the drug-rich particles presumably formed upon dissolution of the ASD, it could be assumed that they are an efficient drug delivery system. This effect seems to also enhance the absorption pharmacokinetics of intervention 1 (ASD of efavirenz 50 mg). However, here the dissolution overall was slow and time to dissolution comparable to the overall transit time of 44 h (including colon transition) used in PK-Sim. Based on the modeled dissolution of intervention 2 (dissolved ASD of efavirenz 50 mg), it could be assumed that this slower dissolution was mainly based on the dissolution of the ASD from solid to liquid (presumably under formation of drug-rich particles). The marketed formulation (efavirenz 50 mg) showed an even slower dissolution in the model, indicating an incomplete dissolution and excretion of undissolved drug substance.

The model has several limitations. Mostly, it can only be partially validated based on the available data due to the lack of an intravenous formulation of efavirenz and therefore the lack of intravenous PK data. With this, bioavailability is unknown and it cannot be estimated if the excretion of undissolved drug in the marketed formulation is feasible. Based on these limitations, intervention 3 (efavirenz solution 3 mg) was the best possible way to calibrate the model. Furthermore, while the model takes into account high protein binding and affinity to adipose tissue of efavirenz, these drug properties are expected to introduce more critical parameters into the model and therefore more uncertainties.

With these limitations, the PBPK model fitting strengthens and further details interpretations made on classical pharmacokinetic analysis alone. It is an attempt to extract most relevant properties of formulation behavior, i.e. intestinal dissolution, from the indirect experimental measurement of plasma concentration -time curves. It highlights, that even small differences in PK profiles could be based on larger differences in dissolution behavior of the formulation. Such analysis of formulation behavior beyond PK profiles could contribute to the development of more complex formulations such as ASDs.

Supplementary Material S2. Dynamic Light Scattering of intervention 2 (dissolved ASD of efavirenz 50 mg)

To characterize the drug-rich particles formed upon dissolution in intervention 2 (compare to 2.3 study interventions), the particle size was measured by dynamic light scattering on a Zetasizer (Malvern Panalytical, Malvern, UK). The ASD of efavirenz was dissolved in a solution of 10 mL of a medical sodium phosphate solution (Colophos®) added to 490 mL of MiliQ Water. The results showed unimodal particle size distribution with a mean particle size of 96.6 nm, thus confirming the presence of drug-rich particles in the drinking solution.

References

1. Langenbucher, F. Letters to the Editor: Linearization of dissolution rate curves by the Weibull distribution. *J. Pharm. Pharmacol.* **1972**, *24*, 979–981, doi:10.1111/j.2042-7158.1972.tb08930.x.
2. Basic anatomical and physiological data for use in radiological protection: Reference values. A report of age- and gender-related differences in the anatomical and physiological characteristics of reference individuals. *Ann ICRP* **2002**, *32*, 5–265.
3. Ke, A.; Barter, Z.; Rowland-Yeo, K.; Almond, L. Towards a Best Practice Approach in PBPK Modeling: Case Example of Develop-ing a Unified Efavirenz Model Accounting for Induction of CYPs 3A4 and 2BCPT. *Pharmacomet. Syst. Pharmacol.* **2016**, *5*, 367–376.
4. Takano R, Sugano K, Higashida A, Hayashi Y, Machida M, Aso Y, et al. Oral Absorption of Poorly Water-Soluble Drugs: Computer Simulation of Fraction Absorbed in Humans from a Miniscale Dissolution Test. Pharm Res. **2006**, *23*, 1144–1156.
